# ‘Suspect molecular signature’ in blood as the indicator of undiagnosed breast cancer, cancer risk and targeted prevention

**DOI:** 10.1186/1878-5085-4-22

**Published:** 2013-09-16

**Authors:** Manuel Debald, Kristina Yeghiazaryan, Melanie Cebioglu, Walther Kuhn, Hans H Schild, Olga Golubnitschaja

**Affiliations:** 1 Department of Obstetrics and Gynaecology, Centre for Integrated Oncology, University of Bonn, Bonn, Germany; 2 Breast Cancer Research Centre, University of Bonn, Bonn, Germany; 3 Department of Radiology, Rheinische Friedrich-Wilhelms-University of Bonn, Sigmund-Freud-Str. 25, Bonn, Germany

**Keywords:** Breast cancer risk assessment, Cancer predisposition, Molecular pattern, Economy, Ethics, Predictive diagnostics, Preventive health care

## Abstract

**Background:**

Breast cancer is a multifactorial disease with the highest incidence rates amongst all cancer types. Further, high levels of circulating tumour cells are a characteristic of breast cancer patients demonstrating a particular predisposition to the development of breast cancer metastatic disease. Actual diagnostic approaches are frequently unable to recognise early stages of tumour development which impairs individual outcomes. In contrast, predictive and preventive risk assessment and early diagnosis may lead to full recovery after surgical resection. Recently, the authors have reported about the construction of diagnostic windows, which could influence the molecular diagnostics of breast cancer.

**Material and methods:**

In a previous study, diagnostic windows for breast cancer risk assessment were analysed. Women with non-malignant breast diseases demonstrating molecular profiles similar to those of breast cancer patients were enrolled into this follow-up study. In the interviews, for patients identified as predisposed to cancer, a specialised questionnaire has been set up to characterise individual risk factors and estimate their potential impacts on cancer onset and progression.

**Results and conclusions:**

By utilising the technological tool of diagnostic windows, 13 individuals have been identified demonstrating molecular profiles typical for patients diagnosed with breast cancer. The current paper summarises the analytical results and makes statements to the application of the pathology-specific molecular profiles recognised as the technological tool for improved diagnostic approach, breast cancer risk assessment and preventive health care management. The necessity to create individual patient profiles and analyse the evolution of the molecular signature is justified for advanced medical services. *Expert recommendations* are provided to promote further developments in the field of advanced breast cancer management.

## Overview

### Breast cancer incidence strongly impacts health, health care and the economy of medical services

The incidence of breast cancer continually increases worldwide during the past three decades. Hence, in the USA, the highest cancer-related incidence rates are currently registered for breast cancer patient cohorts [1,2]. According to the statistical data published by the National Cancer Institute in the USA [3], the estimated new cases and deaths from breast cancer in the USA in 2012 are as follows (in thousand cases):

➢ New cases, 226,870 (female); 2,190 (male)

➢ Deaths, 39,510 (female); 410 (male)

Regarding the economy of corresponding medical services, in the USA, the costs for prescriptions against breast cancer create the second leading category of all pharmaceutical sales with enormously increasing rates [4]. The therapy costs for each patient with metastatic breast cancer have been reported reaching US$128.556 over a mean follow-up time of 18 months [5].

### Circulating tumour cells are most frequent in breast cancer amongst all cancer types

The high level of circulating tumour cells (CTC) secondary to breast cancer onset has been described in the literature to highly predispose the patient cohort to breast cancer metastatic disease (BCMD) [6-8]. Consequently, blood tests are a promising approach for diagnosing BCMD. A further promising diagnostic approach might be the molecular characterisation of CTC as the predictor of tumour invasiveness and therapy response [9].

### Breast cancer metastatic disease is currently incurable

At the time of diagnosis, a great portion of patients with breast cancer have locally advanced and/or distant metastatic disease already. Further, 20% to 30% of patients with early breast cancer will develop relapse with distant metastasis, mainly to the liver, lung, bones and brain [10]. Once breast cancer has turned metastatic, the disease is recognised as an incurable one: the 5-year survival barrier will be reached by only 26% of patients treated for BCMD.

Breast cancer metastasises predominantly into the lymph nodes, bones, lung, skin, brain, and liver [11]. With the poorest prognosis of approximately 80% mortality rate within the first 12 months of diagnosis, brain metastases represent a devastating category of BCMD.

Advanced imaging technologies are currently considered as being the most appropriate tool to diagnose BCMD, to detect primary lesions and to trace distant metastases over the whole body (whole-body imaging). Well-recognised technologies are the multidimensional and multimodal ones: CT, MRI, PET, SPECT and ultrasound; PET and the combined PET/CT are the key tools for whole-body scanning. However, there are some substantial clinical deficits which imaging technologies suffer from [12]. Small-sized metastases in the lymph nodes may be detected by amplification of the smallest amounts of transcripts produced by BCMD biomarkers such as CK19 and others. However, a conclusion might be doubtful, due to untargeted biomarkers, particularly for heterogeneous tumours which is, indeed, the frequent case [12].

### Breast cancer risk assessment

Early detection of the tumour has been demonstrated to be highly beneficial for significantly enhanced therapy efficacy. Approaching predictive diagnosis may lead to full recovery after surgical resection. Furthermore, detection of individual predisposition to breast cancer represents the optimal way to trigger targeted preventive measures before the clinical onset and development of the fatal BCMD. Breast cancer risk assessment is currently extensively under consideration. In a recently published article, a detailed justification of the ‘molecular patterns in activated leucocytes as the minimally invasive diagnostic tool for breast cancer risk assessment’ has been provided by the authors [1]. From the facts and conclusions summarised there, it is getting obvious that the pathology-specific molecular/expressional patterns in orchestrated leucocytes are activated strictly in accordance to the precancerous/cancer stage. Therefore, if detected in correlation with the corresponding disease initiation and progression stage, these patterns in activated leucocytes might be of high relevance for diagnostic and treatment purposes.

This consideration led to the idea of creating a minimally invasive approach for breast cancer risk assessment based on *ex vivo* blood tests by examination of the specific molecular/expressional patterns in circulating leucocytes.

### Construction of diagnostic windows for minimally invasive breast cancer risk assessment based on blood tests

This multimodal approach utilises a combination of conventional analytical methodologies for the creation of pathology-specific biomarker patterns at complementary levels of detection, namely:

– Medical imaging (primary tumour, distant metastasis)

– Sub-cellular/molecular imaging by ‘comet assay’ DNA analysis (risk assessment for general tumour predisposition)

– Clinical differential proteomics as a ‘gene hunting’ approach for pathology-specific molecular patterns in blood cells

– Blood metabolomics for quantification of disease-relevant metabolite patterns

– Quantitative analysis of enzymatic activities in blood plasma

– Others followed by mathematical modelling of pathology-specific profiles

The detailed description of the diagnostic windows constructed for breast cancer risk assessment is provided in our earlier publications [1,13,14].

The current paper is dedicated to the application of the constructed diagnostic windows for the breast cancer risk assessment in a non-malignant (control) group of patients recruited at the Department of Gynaecology, University of Bonn.

## Methods

### Recruitment of patients and blood sampling

In a previous study, 161 patients proportionally distributed between two pools: a group with malignancies (invasive lobular and ductal carcinomas; 82 patients) and a group of ‘non-malignant controls/benign lesions’ (fibroadenomas, fibrocystic diseases, lipomas, adenosis and breast traumas; 79 patients), were recruited at the ‘Breast Cancer Research Centre’, Rheinische Friedrich-Wilhelms-University of Bonn. According to the diagnosis, the recruited patients were grouped as follows: benign breast lesions in pre-menopausal women (group 1, *n* = 59), benign breast lesions in post-menopausal women (group 2, *n* = 20), invasive breast cancer in pre-menopausal women (group 3, *n* = 19) and invasive breast cancer in post-menopausal women (group 4, *n* = 63). Blood samples of all patients were taken prior to the application of any invasive procedure such as a core needle biopsy at the Department of Obstetrics and Gynaecology. All participants were informed about the purpose of the study and correspondingly signed the ‘consent of the patient’. All investigations conformed to the principles outlined in the Declaration of Helsinki and were performed with permission by the responsible Ethics Committee of the Medical Faculty, University of Bonn.

### Diagnostic windows for breast cancer risk assessment

The construction of the diagnostic windows for breast cancer risk assessment was the purpose of the previous study. The detailed description of the technology, as well as the critical analysis of both its advantages and limitations, is provided in a series of our previous issue-related publications [1,13,14]. Herewith, we wish to summarise the entire approach.

#### Quantitative sub-cellular analysis by comet assay imaging

The comet assay provides a simple and effective method for evaluation of DNA damage and DNA repair capacity in single cells such as leucocytes. The principle of the assay is based upon the ability of DNA fragments to migrate out of the cell under the influence of an electric field. An evaluation of the ‘comet’ tail shape and DNA fragments migration pattern allows for assessment of DNA damage and repair capacity. DNA damage is assigned to four classes based on the visual aspect of the comets, considering the extent of DNA migration as published earlier [15]. For breast cancer patients, the disease-specific comet patterns have been characterised [14] as follows:

– Increased damage to DNA

– Debilitated apoptotic reaction towards increased DNA damage

– Pathology-specific comet patterns

– Impact of hormonal status on the specificity of comet patterns amongst breast cancer patients

– Characteristic windows of comet patterns that may be utilised for breast cancer risk assessment—both positive (at high risk) and negative (at low risk) prediction

The constructed comet assay-based diagnostic windows clearly distinguish between the molecular profiles of tumour and benign patients; therefore, they are considered for practical application in differential molecular diagnostics.

#### Breast cancer-specific protein expression patterns

Clinical differential proteomics performed in our previous study revealed breast cancer-specific protein expression patterns [1]. The affected functional groups applied to the pathology-specific diagnostic windows are the following:

1. Microfilamental network-associated and cytoskeletal assembly proteins

2. Cell motility, migration and adhesion

3. Nucleoside/nucleotide turnover and metabolism

4. Protein metabolism (regulatory protein synthesis and protein modification enzymes, chaperons)

5. Energy metabolism

6. Vitamin metabolism

7. Mitochondrial proteins

8. Channels, membrane architecture and intercellular junction proteins

9. Anti-oxidant defence/redox control

10. Detoxification proteins

11. Stress response/protection-related proteins

12. Cell cycle machinery proteins

13. Heat-shock proteins

14. Apoptosis-related proteins/protection against apoptosis

15. Tissue remodelling enzymes

16. Extra-cellular transport and carrier proteins

17. Signal transduction proteins/signalling pathways

18. Longevity/ageing-related proteins

19. Inflammation-related/anti-inflammatory proteins

20. (Breast) cancer-related inhibitors/promoters

21. Cancer invasion and regulators of metastases formation

The complete literature overview considering the functional relevance of the above-summarised protein groups to cancer development and metastatic diseases is provided in our recently published review article [1].

### Application of the diagnostic windows to the collective of patients with benign breast lesions

For the group of patients originally diagnosed as being non-malignant (altogether 79 persons), individual molecular profiling has been performed and its potential specificity for breast malignancy has been evaluated utilising the above-described diagnostic windows. Individual molecular profiles have combined sub-cellular imaging for DNA quality (quantitative comet assay) and characterisation of the stress proteome and microfilamental network-associated proteins in circulating leucocytes. Integrative bioinformatics has been approached to evaluate individual profiles. Patients who have demonstrated molecular profiles similar to those of breast cancer patients were enrolled into the follow-up study. Data on patient history at the time of initial blood collection in 2005 or 2006 were obtained from the original health records. The follow-up was performed after informed consent.

### Follow-up examinations

#### Including criteria

All patients from the pool of non-malignant controls/benign lesions but demonstrating molecular profiles similar to the profiles of breast cancer patients have been invited to participate in the follow-up study.

#### Excluding criteria

Patients with malignant breast tumours at the time of original diagnosis have been excluded from the follow-up study.

#### Specialised questionnaire

A specialised questionnaire was created in order to collect information which might provide insights into the reasons of the ‘suspect molecular signature’ in terms of its high similarity to the molecular profiles characteristic of the breast cancer patient cohort. Previous and current data on medical history for each patient and their families were collected using the following questions:

1. Did you suffer from any other disease in 2005/2006 (high blood pressure, asthma, chronic pain syndrome, diabetes, etc.)?

2. Are you currently suffering from any illness that has not been diagnosed in 2005/2006 but later on?

3. Have you ever been diagnosed cancer?

4. Do your close relatives suffer from cancer or any other chronic diseases (diabetes, asthma, etc.)?

5. Did you suffer from any kind of stress (familiar, occupational, etc.) in 2005/2006 or did you suffer from a high degree of psychological strain due to uncertainty of the histological results?

6. Did you smoke in 2005/2006?

7. Do you smoke right know?

8. Do you have children?

9. Have you been pregnant in 2005/2006?

10. Are you pregnant?

#### Communication/interviews with patients

Each patient was sent a questionnaire by mail together with an invitation for the follow-up study to be performed in our clinic. In case we did not receive any response, patients were contacted by a phone call, and the form was filled in by phone interview.

## Results

### Identification of patients with suspect molecular signature

The technological tool ‘diagnostic windows for minimally invasive breast cancer risk assessment based on immune cells profiling’—the previously detailed ‘know-how’ of the authors [1], is a multimodal approach utilising pathology-specific biomarker patterns.

In the current study, the two complementary levels of detection are as follows:

1. Sub-cellular imaging by quantitative (DNA) comet assay analysis.

2. Differential proteomics of the targeted functional groups (‘stress proteome’ and microfilamental network-associated proteins—both highly relevant for cancer onset and metastatic disease) have been applied to 79 blood samples (circulating leucocytes) of non-malignant female patients for individual profiling and comparative analysis with breast cancer characteristic patterns.

Altogether, 13 patients have been identified as demonstrating molecular profiles similar to those of breast cancer patients. These selected patients were included into the follow-up examinations, which was the focus of the current study.

### A revision of the original health records: 3 breast malignancies revised amongst 13 patients with suspect molecular signature

Consequently, a revision of the originally performed health records resulted in the awareness that 3 from the 13 patients demonstrating a *suspect molecular signature* were misleadingly enrolled into the group with non-malignant controls. The patient and tumour characteristics of the three patients with an invasive breast cancer at the time of first diagnosis are summarised in Table 1. For the tumour marker CA15-3 (43.7, 2.0 and 31.1 U/ml), all the patients demonstrated a negative blood level (by definition, 0–53 U/ml) prior to surgery.

**Table 1:** Patient and tumour characteristics of patients with invasive breast cancer at the time of initial diagnosis

**Tumour number**	**Age**	**Menopausal stage**	**Histology**	**T**	**N**	**M**	**G**	**ER**	**PR**	**Her2/neu**
1	46	Peri-menopausal	Invasive ductal breast cancer	1c	0	0	2	Positive	Positive	Negative
2	42	Pre-menopausal	Invasive ductulo-lobular breast cancer	1c	2a	0	3	Positive	Positive	Negative
3	63	Post-menopausal	Invasive ductulo-lobular breast cancer	1c	0	0	2	Positive	Negative	Negative

*ER* estrogen receptor, *PR* progesterone receptor.

**Figure 1:**
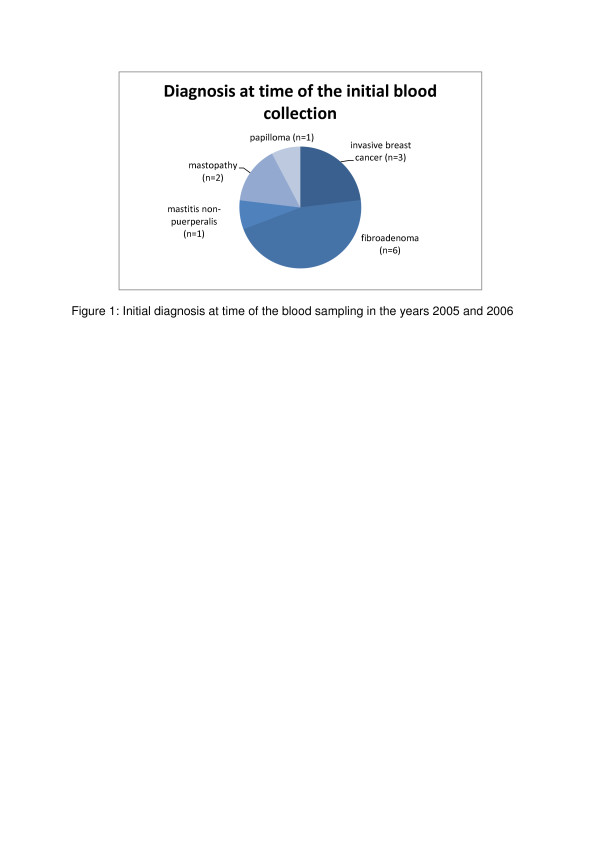
Initial diagnosis at time of the blood sampling in the years 2005 and 2006.

The remaining ten patients were correctly classified as *currently non-malignant* ones. Figure 1 provides a prospective diagnosis overview at the time of blood sampling in the years 2005 and 2006.

Ten patients with a benign histology were invited to participate in the follow-up study. However, solely seven patients were able to participate and have returned the questionnaire or were contacted by a phone call. All seven patients, who underwent the follow-up breast examination either by mammography or ultrasound, have not showed any evidence of malignancy in the breast.

### Benign breast alterations

The characteristics of patients with a benign breast alteration are summarised in Table 2.

**Table 2:** Characteristics of the patients with non-malignant breast alterations selected for the follow-up study

**Patient number**	**Age**	**Menopausal stage**	**Histology**
1	29	Pre-menopausal	Fibroadenoma
2	37	Pre-menopausal	Fibroadenoma
3	31	Pre-menopausal	Fibroadenoma
4	53	Post-menopausal	Mastopathia
5	51	Post-menopausal	Papilloma
6	43	Pre-menopausal	Fibroadenoma
7	51	Post-menopausal	Mastopathia
8	68	Post-menopausal	Mastitis non-puerperalis
9	39	Pre-menopausal	Fibroadenoma
10	44	Pre-menopausal	Fibroadenoma

### Breast tissue inflammation

One 68-year-old patient was diagnosed with a lymphocytic mastitis non-puerperalis in the histological workup of the breast biopsy. This patient reported a palpable mass in her right breast. At the time of biopsy, there was no evidence of a systemic inflammation.

### Non-cancerous chronic diseases

In order to reveal further insights into potential effectors that could have led to an alteration of the molecular signature, secondary diagnoses were evaluated. Table 3 summarises the secondary diagnoses at the time of initial blood collection (*n* = 7). One patient underwent partial vulvectomy due to vulvar intraepithelial neoplasia grade III shortly prior to the blood analysis.

**Table 3:** Secondary diagnosis at the time of initial blood collection

**Secondary diagnosis**	**Number**
Healthy	2
Chronic inflammation	1
Cardiac arrythmia	1
Addison's disease	1
Chronic pain syndrome	1
Vulvar intraepithelial neoplasia grade III	1

In addition, the onset of further secondary diagnoses in recent years was asked for. It turned out that one of the healthy patients developed a ‘burnout’ syndrome, whereas the patient with chronic inflammation got chronic heart disease due to atherosclerosis. None of the patients developed a malignancy at the time point of the follow-up examination.

### Pregnancy

One 31-year-old patient with fibroadenoma was pregnant (second trimester) at the time of initial blood collection. In this benign case, the pregnancy may have caused the alteration of the molecular signature.

### Familial predisposition to cancer is highly pronounced in the group selected for the follow-up examination

Since breast malignancy may be associated with a familial predisposition, a cancer-related family history has been evaluated for each patient in the selected group. Indeed, the family predisposition to cancer has been estimated as significantly higher in the group compared to the general population, since each patient from the individuals selected for this study had at least one first- or second-degree relative with a cancer history. The associated malignancies are summarised in Table 4. Further, one patient has been tested positive for BRCA1 specific mutations.

**Table 4:** Family history regarding cancer

**Cancer type**	**Relative**	**Number of patients**
Breast cancer	Mother	2
	Sister	1
	Grandmother (maternal)	2
	Aunt (paternal)	3
Ovarian cancer	Grandmother (maternal)	1
Colorectal cancer	Grandfather (maternal)	1
	Grandaunt (maternal)	3
Prostate cancer	Father	1
	Uncle (paternal)	1
Bronchial carcinoma	Grandfather (maternal)	1
Adenoid squamous cell carcinoma	Father	1
Glioblastoma	Father	1

### Diabetes history in the family

Since diabetes mellitus is acknowledged as a strong risk factor for malignancies developed secondary to metabolic disease, our questionnaire requested for corresponding information. Consequently, three patients pointed out that there is a cluster of diabetes type 2 in the family.

### Estimation of the stress impacts

Palpable breast alterations cause deep anxiety and stress reactions of the affected individuals. Pronounced stress may result in a systemic body reaction leading to the dramatically altered molecular profiles. In order to estimate potential impacts of stress factors, patients have been asked for their stress-relevant experience and condition at the time point of the original diagnosis/blood sampling. Patient statements regarding stress condition and anxiety towards diagnosis are summarised below:

– ‘…fear of having cancer’

– ‘…was afraid of getting breast cancer during my pregnancy’

– ‘…familial stress and stress at work’

– ‘…occupational stress in the work place’

– ‘…fear of getting cancer (such as many family members)’

– ‘…excessive pressure’

### Smoking habits

A strong predisposing risk factor for the cancer-related alterations of molecular profiles is smoking which has been evaluated within current study. At the time of original diagnosis/blood sampling, one patient used to smoke. At the time of the follow-up study, nobody from the selected individuals has changed original smoking habits.

### Results interpretation

In recent years, multiple validated biomarkers have made their way into clinical routine in the field of breast cancer management [1]. Most of these factors may help clinicians to get insight into patient outcomes or give predictive information about optimal therapy regimes. However, those markers share the similarity of giving information for women only after the onset of breast cancer.

Management of breast cancer has a dramatic influence on health care and economic consequences. In the actual context of extensive debates concerning increasing costs of medical services and limited resources to cover health care costs, new strategies need to be applied towards more effective breast cancer management. To give an example, in the USA, the costs for prescriptions against breast cancer are the second largest category of all pharmaceutical sales with enormously increasing rates [4]. The therapy costs for each patient with metastatic breast cancer have been reported to be US$128.556 over a mean follow-up time of 18 months [5], but also the trend towards the detection of earlier breast cancer stages is under discussion regarding treatment savings. The savings of costs for treatment and palliative care in advanced breast cancer may be counterbalanced by the high costs of more aggressive initial treatments and longer follow-up [16,17]. The recommended screening by clinical breast examination and mammography is able to detect breast cancer in early stages and has been shown to reduce mortality [18-20]. Nevertheless, this screening procedures cause relatively high percentages of both false-negative and false-positive results that lead either to undiagnosed malignancy or to overdiagnosis and overtherapy, respectively [21-23]. Further, a relatively low tumour size sensitivity, mainly in young women (<50 years) with dense breast parenchyma, produces a big portion of scepsis regarding real benefits by mammography screening [24]. Due to its detection limitations, mammography results in the correct diagnosis of breast malignancies ranging from 68% to 90% cases in women aged 50 years and older. In contrast, the detection sensitivity is much lower in women aged 40–49 years with estimated detection rates ranging between 62% and 76% [25]. Likewise, a meta-analysis of randomised controlled trials showed a decreased mortality reduction of only 15% in young women (39–49 years) undergoing regular screening by mammography, compared to 30% in women aged 49–59 years [26]. Therefore, established mammography screening programmes are mainly addressed to older women (50–69 years) [27]. In addition, screening by mammography is cost intensive, restricted to specialised screening units and not feasible in most economically developing countries [28]. Thus, the promotion of awareness of early breast cancer signs and symptoms as well as sole clinical breast examination remains the only early detection strategies for low-income and middle-income countries [29].

As demonstrated above, recent studies of our group led to the creation of diagnostic windows for breast cancer risk assessment by molecular profiling and sub-cellular imaging demonstrating individual predisposition to breast malignancies [13,14]. This is the next step forward towards predictive medicine. The present findings suggest that the use of pathology-specific molecular profiles is a feasible approach for individualised breast cancer prediction. With the current analysis, we have shown that the suspect molecular signature detected in the ‘control non-malignant group’ was associated with:

1. Breast cancer (3 cases revised for breast cancer from 13 patients with suspect molecular signature)

2. General (breast) cancer predisposition (familial predisposition to cancer is highly pronounced in the group selected)

3. A number of risk factors acknowledged as strongly promoting (breast) cancer development (see data provided above)

4. Other factors influencing the patient's health and mind (stress, etc.)

Obviously, the pathology-specific molecular/sub-cellular profiling approach is a powerful instrument that allows for more precise examination of the level of individual health and that of disease predisposition. Large-scale follow-up studies should be essentially performed in order to:

(a) Evaluate the predictive power of diagnostic windows applied here

(b) Estimate the scale of potential economical benefits of the targeted prevention in groups of risk followed by the creation of innovative economical models for advanced medical services in breast cancer management

(c) Elaborate the ethical aspects of individual prediction

(d) Develop the optimal approaches of participative medicine as an effective form of individualised predictive medicine versus reactive disease care

(e) Find significant correlations for the above-mentioned factors or further factors that have not been evaluated yet

## Expert recommendations

### Innovation by molecular patient profiling

Further prospective studies are necessary to verify our initial results in a larger cohort of patients. Such a study could consist of regular blood analysis in combination with examinations by medical imaging as well as the systematic evaluation of personal and health data at several time points in a group of women with no history of breast cancer such as patients with benign breast diseases and healthy individuals with a family history of cancer, etc. This setting would allow us to get deeper insights into the marker profile of non-cancerous patients and might help identify individual factors relevant for pathology development. Moreover, specific alterations associated with carcinogenesis might be measured at the initiating stages well suited for targeted preventive measures. This action is in a good consensus with the new paradigm proposed to shift health care from reactive to predictive medicine. This approach may lead to the following promising measures in advanced breast cancer management:

1. A molecular profile that shifted towards a pathology-specific signature would justify a need to take action for a deeper and more targeted breast imaging resulting in diagnosing non-invasive forms of breast cancer—the so-called ductal carcinoma *in situ* and lobular carcinoma *in situ*, with excellent survival rates as recorded worldwide.

2. Estimation of the individual breast cancer risk may be realistically performed utilising the regular examination of the molecular profiles. In the case of negative dynamicity, corrective treatment algorithms may be created according to the individual risk factors and affected molecular pathways.

### Potential economical impacts of molecular diagnostics

Economical burden of breast cancer management are permanently increasing, negatively impacting the health care budgets. In contrast, innovative approaches proposed by the paradigm of Predictive, Preventive and Personalised Medicine (PPPM) promote an economically more attractive scenario for investment in health care; however, they should get carefully analysed through well-designed pilot projects. The costs related to the blood-based screening linked to the follow-up diagnostic measures need to be compared with the existing screening methods by mammography from viewpoints of health- and economy-related long-term outcomes. To give an example: A patient is diagnosed breast cancer at a progressive stage. In this case, the patient has to undergo surgery, chemotherapy, radiotherapy and endocrine treatment for several years causing the so-called direct costs for the overall medical care. However, the indirect costs might be even higher, since this patient is not able to accomplish the work anymore or for a long period of time and/or not able to achieve a full-time equivalent becoming strongly handicapped in both professional and social activities as well as the family-relevant ones.

### Ethical considerations

Next to the economical impacts, not less important are also the ethical aspects of the paradigm shift proposed by the PPPM approaches. Currently, patients come to the clinic due to palpable masses in the breast or alarming imaging results. In this paper the results of our analysis demonstrate that the majority of the interviewed patients strongly suffer from anxiety in the time frame essential for the biopsy performance and consider this step as extremely stressful. How will the matter work in the case of providing a predictive diagnosis? Nobody can answer this question, since psychological treatment approaches are completely underdeveloped in the new field of predictive medicine. Especially, the ethical aspects of participative (tight and harmonic collaboration between patient and treating person/s) medicine is a delicate field which is highly relevant for the overall success in health and disease management. The corresponding principles of PPPM ethics should be thoroughly elaborated and approved under controlled conditions in multidisciplinary studies.

### Outlook

Predictive medicine is an innovative approach towards a new era in breast cancer prevention. Breast cancer risk assessment aims at detection of pre-malignant stages that will revolutionise breast cancer management as a whole from the viewpoint of the professional set-up, treatment regimens, life quality of a patient, economy and ethics of medical care. The authors are aware of potential risks linked to any paradigm shifting. Medical care being conservative by definition, on the other side, should essentially progress towards reasonable innovation benefiting patients and health care as a whole. The authors intend to follow the above-justified strategy by setting up large-scale projects with the relevant multidisciplinary expertise to approve the feasibility and credibility of PPPM-related innovation in breast cancer management.

## Competing interests

The authors declare that they have no competing interests.

## Authors’ contributions

MD supervised the patient recruitment and data collection at the Department of Obstetrics and Gynaecology. OG and MD created the concept of the project, made the data interpretation and drafted the article. KY and MC carried out the molecular biological studies. WK supervised the project at the Department of Obstetrics and Gynaecology. HS supervised the project at the Department of Radiology. All authors read and approved the final manuscript.
